# Multifunctional Gold Nanorod for Therapeutic Applications and Pharmaceutical Delivery Considering Cellular Metabolic Responses, Oxidative Stress and Cellular Longevity

**DOI:** 10.3390/nano11071868

**Published:** 2021-07-20

**Authors:** Seyyed Mojtaba Mousavi, Seyyed Alireza Hashemi, Sargol Mazraedoost, Khadije Yousefi, Ahmad Gholami, Gity Behbudi, Seeram Ramakrishna, Navid Omidifar, Ali Alizadeh, Wei-Hung Chiang

**Affiliations:** 1 Department of Chemical Engineering, National Taiwan University of Science and Technology, Taipei 10617, Taiwan; mousavi.nano@gmail.com; 2 Nanomaterials and Polymer Nanocomposites Laboratory, School of Engineering, University of British Columbia, Kelowna, BC V1V 1V7, Canada; sa_hashemi@sums.ac.ir; 3 Biotechnology Research Center, Shiraz University of Medical Sciences, Shiraz 71345-1583, Iran; sargol.mazraedoost7@gmail.com (S.M.); khadije.yousefi@gmail.com (K.Y.); omidifar@gmail.com (N.O.); 4 Pharmaceutical Sciences Research Center, Shiraz University of Medical Sciences, Shiraz 71345-1583, Iran; 5 Department of Chemical Engineering, University of Mohaghegh Ardabili, Ardabil 56199-11367, Iran; gitybh@gmail.com; 6 Department of Mechanical Engineering, Center for Nanofibers and Nanotechnology, National University of Singapore, Singapore 117581, Singapore; seeram@nus.edu.sg; 7 Department of Pathology, School of Medicine, Shiraz University of Medical Sciences, Shiraz 71345-1583, Iran; 8 Nanobiology and Nanomedicine Research Center, Shiraz University of Medical Sciences, Shiraz 71345-1583, Iran; alializadeha@gmail.com; 9 Department of Tissue Engineering and Applied Cell Sciences, School of Advanced Medical Sciences and Technologies, Shiraz University of Medical Sciences, Shiraz 71345-1583, Iran

**Keywords:** multifunctional gold nanorods, therapeutic applications, cellular metabolic responses, oxidative stress, cellular longevity

## Abstract

Multifunctional gold nanorods (GNR) have drawn growing interest in biomedical fields because of their excellent biocompatibility, ease of alteration, and special optical properties. The great advantage of using GNR in medicine is their application to Photothermal therapy (PPTT), which is possible thanks to their ability to turn luminous energy into heat to cause cellular hyperthermia. For this purpose, the relevant articles between 1988 and 2020 were searched in databases such as John Wiley, Free paper, Scopus, Science Direct, and Springer to obtain the latest findings on multifunctional gold nanorods for therapeutic applications and pharmaceutical delivery. In this article, we review recent progress in diagnostic and therapeutic applications of multifunctional GNR, highlighting new information about their toxicity to various cellular categories, oxidative stress, cellular longevity, and their metabolic effects, such as the effect on the energy cycles and genetic structures. The methods for the synthesis and functionalization of GNR were surveyed. This review includes new information about GNR toxicity to various cellular categories and their metabolic effects.

## 1. Introduction

**Figure 1:**
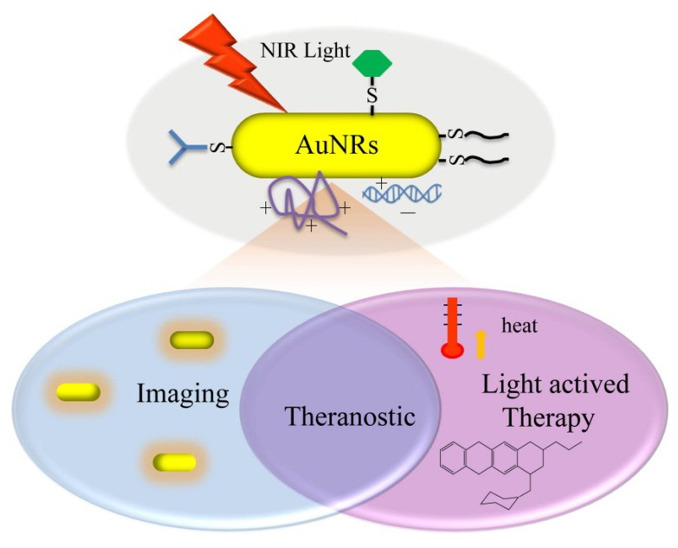
Illustration of different beneficial uses of GNR with light mediation [6]. Reprinted with permission from ref. [6]. Copyright 2013 Theranostics.

Today, nanotechnology scientists have acquired the practical ability to create nanoscale gold particles that have proved to be a “magic tool” in the fight against human diseases. Owing to their extraordinary capacity to capture and scatter light, these gold nanostructures, such as nanospheres, nanorods, nanoshells, nanostars, and nanocages, are of tremendous importance in bio-imaging and disease therapy. Nanorods are defined as structures in nanoscale whose length is two to twenty times longer than their width [1]. There are several reasons why nanorods are relatively superior to spherical particles [2]. Relative to most other types of nanostructures, gold nanorods (GNR) have more significant extinction coefficients and narrower line widths, with higher photothermal conversion efficiencies and better sensitivity to local dielectric constant shifts [3]. These properties have given rise to various exciting possibilities to use GNR for Near-Infrared Resonant (NIR) biomedical imaging methods such as Two-photon lithography (TPL), Photographic Activity Test (PAT), Optical Coherence Tomography (OCT), and X-ray computed tomography (X-ray CT), and for hyperthermic therapy and gene/drug delivery [3,4]. GNR is a platform that allows for mutual understanding of both diagnosis and clinical care in a single program with various diagnostic and therapeutic modalities [5]. Such programs are based on the principle of theranostics, therapy fusion, and diagnostics to maximize the efficiency and health of therapeutic regimes [6] (Figure 1). Theranostic based on GNR can regulate all practical modalities with excellent spatial and temporal precision, which is a significant advantage over other theranostic systems.

Consequently, GNR has proved promising in various biomedical applications, such as imaging, hyperthermic therapy, and drug delivery, due to their flexible surface plasmon and photothermal effects [7,8,9]. These applications can be managed remotely by Near Infrared (NIR) light, penetrating deep into human tissue with limited lateral invasion [10]. GNR, therefore, can integrate medical therapy and clinical treatment into a single framework and act as a theranostic NIR light-mediated network. One instance of the value of rod-like nanostructures was developed by Alivisatos and collaborators [10]. Therefore, the increase in external quantum efficiency is achieved by joining nanorods in poly (3-hexylthiophene) (P3HT) thin films, because the ratio of the problem has been increased from 1 to 10. As the particle ratio increased, the electron concentration also improved. Different work has been carried out to assess the cytotoxicity of GNP due to different surface modifications in human dermal fibroblasts. However, no definitive results have been recorded.

In this paper, the relevant articles between 1988 and 2020 were searched in databases such as John Wiley, Free paper, Scopus, Science Direct, and Springer to obtain the latest findings on multifunctional gold nanorod for therapeutic applications and pharmaceutical delivery. For this goal, cellular metabolic responses, oxidative stress, and cell longevity are also considered. Keywords such as multifunctional gold nanorods, therapeutic applications; cellular metabolic responses; oxidative stress; and cellular longevity are used to search the mentioned databases. Therapeutic applications of multifunctional GNR in Photothermal therapy (PPTT), cancer therapy, and antibacterial activity are discussed; also, the role of GNR in novel delivery systems such as drug delivery vehicles and gene delivery have been studied. Significant results such as GNR cellular metabolic responses and adverse biological effects, multifunctional GNR in cellular longevity, and oxidative stress are mentioned. The articles used included the full length of review articles, abstracts of review articles, and abstracts of original articles that most of the articles used were review articles.

**Figure 2:**
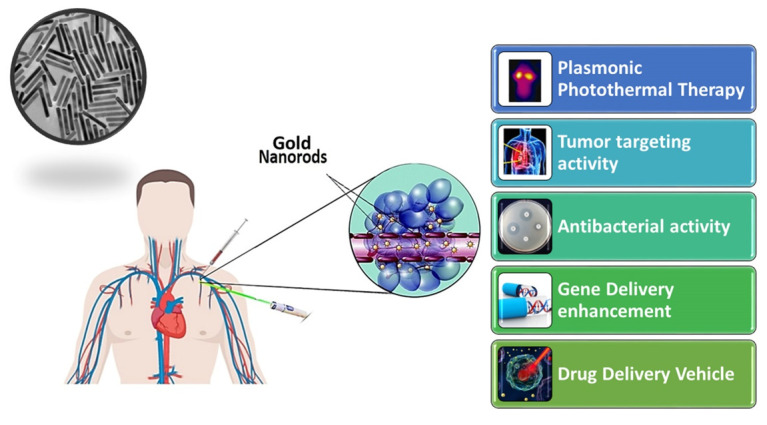
Schematic Illustrations of Nanorod application.

In this article, the methods for the synthesis and functionalization of GNRs were discussed. The applications for hyperthermia therapy, such as drug delivery vehicles and new information about their toxicity to various cellular categories and their metabolic effects, for instance, the effect on the energy cycle and genetic structure, were reviewed (Figure 2).

## 2. GNR Characteristics

**Figure 3:**
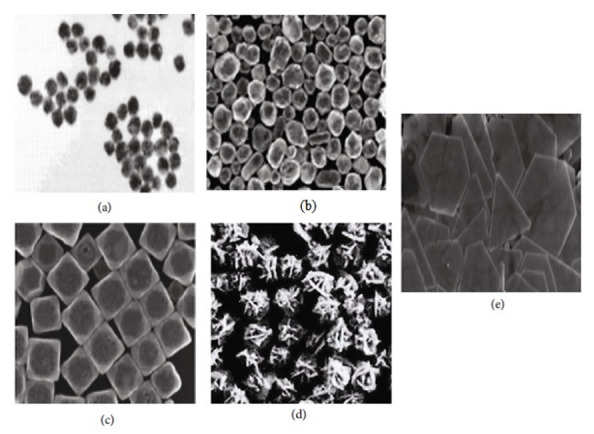
(**a**) Au nanospheres [11]; (**b**) Au nanoshells [12]; (**c**) Au nanocubes [13]; (**d**) Au nanoflowers [14]; (**e**) Au nanotriangles [15]. Reprinted with permission from ref. [11,12,13,14,15]. Copyright 2002,2004,2008 The Journal of Physical Chemistry B, Chemical Communications, Analytical Chemistry, Journal of the American Chemical Society, Langmuir, Springer Nature.

Gold nanomaterials have drawn the interest of material scientists, biomedical scientists, and physicists. These inert nanostructures are gold-based, including typical nanomaterial properties such as surface effect, small size effect, macroscopic quantum tunneling effect, and quantum size effect. Their unique morphology-based optical properties have been extensively used in biosensors, bioimaging, optical information storage, and catalysis. Gold nanomaterials with different morphologies have been synthesized (Au nanospheres [11], Au nanoshells [12], Au nanocubes [13], Au nanoflowers [14], and Au nano triangles [15]) as seen in (Figure 3).

**Figure 4:**
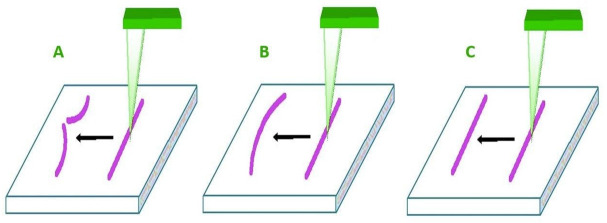
Three possible impacts: (**A**)—rigid nanorod, (**B**)—fluid nanorod, (**C**)—elastic nanorod [16]. Reprinted with permission from ref. [16]. Copyright 2012 Open access peer-reviewed chapter.

A variety of publications and papers provide comprehensive studies of the optical characteristics of metal particles, including electromagnetic theory, size effects, and light scattering. Textbooks giving thorough treatments include those by van der Hulst [5], Kerker [6], and Bohren and Huffman [7]. More recent work has involved developing robust numerical solutions to the light scattering and extinction by non-spherical structures, including 2D arrays [8]. The review by Kelly et al. provides an excellent overview [9], while several authors cover computational aspects. Biomedical uses of GNR-based nano-carriers are depicted schematically. Biologicals and metallics are examples of nanorods found in nature and industry. There are many demands for using the nanorods manipulation depending on how the nanowire is positioned on the example substratum (Figure 4A–C) [16]. Flexible particles with significant deflection can be pushed, but delicate particles may be harmed if the force hits the stress.

Several published research studies have looked into GNP’s interaction with the skin of various shapes, sizes, and surface changes [17,18,19]. Non-spherical GNP has more than a few benefits, owing to their remarkable photothermal and antibacterial activity such as completed spherical equivalents in skin nanomedicine, skin infection treatment and prognosis, wounds and pores, and skin cancer [20,21,22]. The evaluation of cytotoxicity and non-circular absorption of GNP cells, such as GNR in human dermal fibroblast treatment, has been strongly related in the literature. Pernodet et al. exposed to citrate-covered GNP as the final product of their high absorption of cells, induced significant improvements in the human dermal fibroblast cell exercises [23]. Similarly, cytotoxicity was caused by oxidative stress in human dermal fibroblasts, and it was independent of the size of nanoparticles in spherical GNP application [24].

## 3. GNR Synthesis

**Figure 5:**
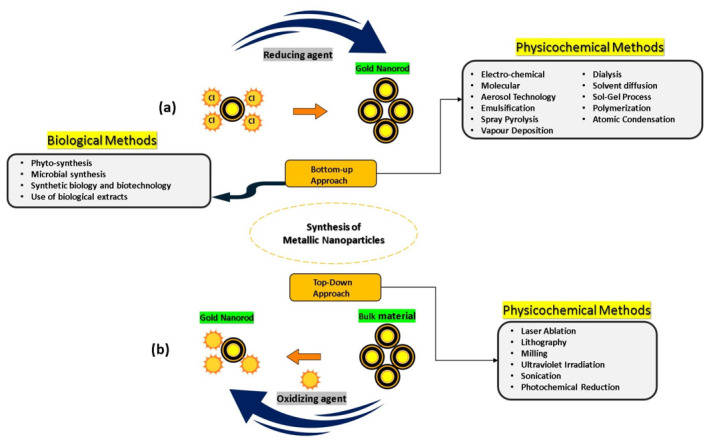
Strategies the synthesis of GNR using traditional physiochemical and relatively novel eco-friendly biological methods (**a**) from the bottom-up and (**b**) top-down approaches.

Au nanorods can be synthesized by two different methods of production, which are bottom-up and top-down strategies (Figure 5). Au nanorods are created via nucleation in aqueous and related overgrowth procedures for bottom-up approaches such as microwave-assisted, electrochemical, sonochemical, wet-chemical, solvothermal, and photochemical decline counts. Au salts are commonly utilized to minimize the Au base. This method, including the use by various decreasing operators of reduced watery, solved Au salts, for instance, ascorbic corrosive, sodium borohydride, and small Au clusters, under particular environmental conditions (triggering the reduction of Au salt). The primary method for producing the electrochemical approach of Au nanorods was short. Au and Pt were used as anode and cathode, principally. These anodes can be dissolved in a solution containing a cationic surfactant such as hexadecyltrimethylammonium bromide (CTAB) and a co-surfactant. The length of the nanorods is measured by the position within the structure of a silver plate. The silver metals react to the particles made by the decay of the anode. Typically, the tiny Au nanoparticle seed of 1.5 nm is initially arranged in a watery CTAB arrangement by decreasing chloroauric corrosive with borohydride. The seed arrangement will be combined with production arrangements containing metal salts such as corrosive ascorbic (powerless lessening specialist) and CTAB, a specialist in surfactant-steering. The CTAB will bind to Au nanorods and will shape a bilayer.

The seed-mediated progress method is close to CTAB, and it can produce nanorod yields of up to 99%. It has been determined what kind of Au it is. Consequently, gold nanorods have the probability of being used as thermal therapeutic agents for selective damage to cancer cells, bacterial cells, viruses, and DNA. The user may tune the aspect ratios and equivalent LSPR optical peaks of gold nanorods grown by colloidal seed-mediated growth with the possibility of easy volumetric synthesis scaling. However, this adaptability has downsides since it demands precise control of various factors in various stages of the process. Because of the large number of variables, maintaining response uniformity and repeatability is difficult. Researchers aiming for large-scale in vivo investigations may become frustrated as a result of this.

**Figure 6:**
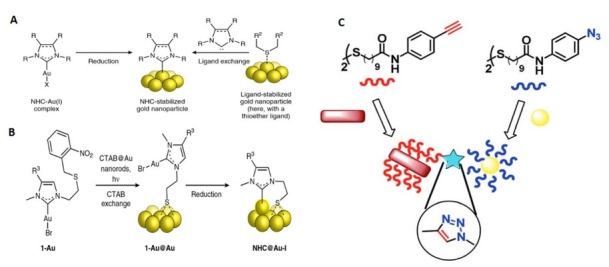
Strategies for NHCs being based on gold nanomaterials (**A**) Previous methods involve the reduction of preformed or in situ generated (the latter from imidazolium tetrahaloaurate salts) NHC–metal (for example, gold) complexes, or the displacement of weakly bound (for example, thioether or amine) ligands with free NHCs. X, a coordinating ligand, typically a halogen such as Cl or Br; R and R2, alkyl, aryl or other substituents. (**B**)Here, we introduce a bidentate thiolate masked NHC strategy whereby exchange of CTAB ligands on commercial CTAB@Au nanorods with a photogenerated thiolate is followed by NHC installation (**C**) new surface-bound NHC–gold adatom complex [44]. Reprinted with permission from ref. [44]. Copyright 2018 Nature Chemistry.

In the case of NHC (NHC stands for Non-Histone Chromosomal proteins), surface chemistry, the swapping of CTAB or citrate from industrial nanorods with a suitable NHC would be appropriate; however, current ligand exchange methods (Figure 6A) involving the production of free NHCs are unlikely to be effective in aqueous solutions using ligands such as CTAB or citrate [25,26,27,28,29,30,31]. In addition, direct reduction methods for the synthesis of NHC-stabilized nanoparticles have provided only relatively small nanomaterials (15 nm), which are strictly spherical (Figure 6A) [31,32,33,34,35,36,37,38,39]. However, mounting NHCs on gold nanomaterials of any random size and form requires a particular technique. Here we pose an approach to delivering bidentate NHC–thiolate ligands to gold nanorod surfaces overcoming the above limitations and yielding robust nanorods (NHC@Au nanorods, Figure 6). Their strategy leverages the well-established exchange of thiolate ligands on CTAB-stabilized GNR [40] to carry masked NHCs near the surface of the nanorod . Their approach has been to transport masked NHCs near the nanorod surface [41] for stabilized exchange of thiolate ligands in the GNR [40]. The original nanorod size and shape retained in NHC@Au nanorods indicated limited nanorod restructuring from the ligand addition, because installing NHCs by decreasing NHC–gold (i) complexes linked to the nanorod surface would produce stability [30,40,41].

Nevertheless, here they [42,43] prove that this process of ‘atomization’ (Figure 6B) contributes to stable NHC@Au nanorods under a wide variety of harsh conditions, as well as those where conventional NHC and thiol@Au nanorods are unstable. In addition, assays to culture in vitro cells indicate that NHC@Au nanorods are promising materials for laser-induced Partial thromboplastin time (PTT). In this way, the two nanostructures, which reacted with 1,3-dipolar click cycloaddition between acetylene on GNR and an azide group on the silver nanoparticles, are likely to be covalently connected (Figure 6C) [44].

**Figure 7:**
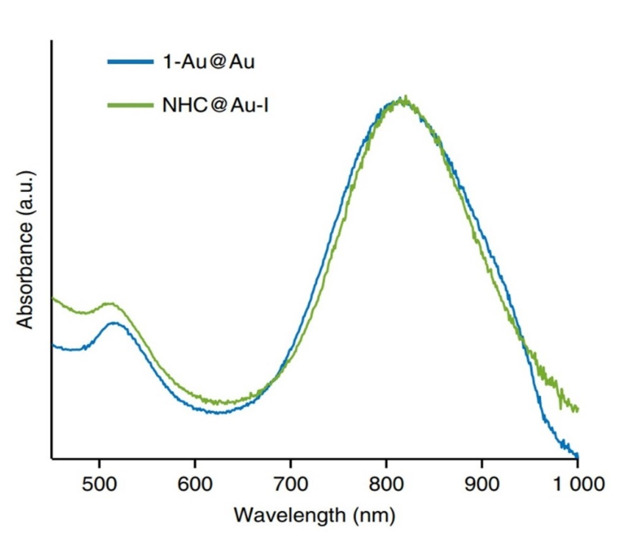
UV—vis spectra for 1-Au@Au andNHC@Au-i indicate that the size and shape of the nanorods are preserved when transforming 1-Au@Au to NHC@Au-i (i.e., adatom incorporation does not have a dramatic effect on nanorods optical properties) [44,45]. Reprinted with permission from ref. [44,45]. Copyright 2018 RSC Advances.

Previous methods include the reduction of NHC-metal complexes (for example, gold) or the displacement of weakly bound ligands (for example, thioether or amine) with free NHCs in preformed or in situ produced (the latter from imidazolium tetrahaloaurate salts). Using meth-188 criteria, it has not been possible to install NHCs on gold nanomaterials with random sizes and shapes. The synthesis of 1-Au@Au thiolate monolayer from 1-Au photoprotection and its subsequent reduction to NHC@Au-i production introduces a new NHC-gold adatom complex on the surface (Figure 7). The resulting stable GNR of bidentate thiolate–NHC have the same size and form as their industrial parent nanorods, and they are resilient against various strict conditions [44,45].

## 4. GNR Functionalization

Nanomaterials for medical applications require proper functionalization to have biocompatibility and/or recognized properties in a biological environment. CTAB creates a bilayer on the gold nanorod surface, and the repulsion in the quaternary cationic ammonium head has implications in steady GNR in aqueous media [46]. However, CTAB stabilized GNR in serum, and phosphate-buffered saline (PBS) is not constant, with or disadvantaged from media, which may restrict their usefulness in biological applications [47]. In addition, CTAB is obstructive and blocks surface modification access with bioconjugates [48,49]. To this end, many techniques have been investigated to substitute CTAB on nanorod surfaces for biomedical applications or for overcoming CTAB toxicity. One method requires the removal of CTAB from GNR suspensions into chloroform phases containing phosphatidylcholine (PC). Compared to CTAB GNR, the PC-modified GNR presents low cytotoxicity [48,49]. Another option is to use layer-by-layer polyelectrolyte deposition on nanoparticle surfaces to enhance the stability of GNR [50].

The above systems have demonstrated excellent PBS and serum stability, thus maintaining the optical properties. Nevertheless, there is concern about the negative effect of CTAB on the resonance of surface plasmon and the additional coatings. Tian et al., for example, observed that the duration of the electromagnetic decay increases linearly for both nanorod duration and diameter for these polyelectrolyte-functionalized NRs. This is even more important for applications that use GNR for sensors and detectors. Given their high binding affinity to gold, alkanethiols were widely used to replace CTAB molecules [51,52]. The above systems have demonstrated excellent PBS and serum stability, thus maintaining the optical properties. However, some concern remains about the negative effect of CTAB on the resonance of surface plasmon and the additional coatings. Tian et al. observed that for both nanorod duration and diameter for these polyelectrolyte-functionalized NRs the time of the electromagnetic decay increases linearly. This phenomenon is even more important for applications that use GNR for sensors and detectors. Given their high binding affinity to gold, alkanethiols have been widely used to replace CTAB molecules [53]. In this case, N-methyl formamide is extracted from the CTAB and combined with phospholipid-dextran. This method displays flexibility over a variety of values for pH, salt conditions, and serum addition. Even CTAB can be substituted with thiolated ligands [54]. GNRs coated with polyethylene glycol (PEG) are commonly used for biological applications. It is because PEG was discovered to increase nanomaterial circulation time [53] and new formulations [55,56]. Von Maltzahn et al. found that PEG-coated GNR had high stability, relative in vitro non-toxicity, and large-circulation that permits their passive tumor accumulation [57]. Numerous strategies have been informed for the functionalization of PEG on GNR [58,59,60,61]. PEG replaces CTAB through thiolization via a strong sulfur metal bond [62].

## 5. Therapeutic Applications of Multifunctional GNR

### Photothermal Therapy (PPTT)

Several studies have shown GNR, as therapeutic agents, directly target malignant cells, while healthy cells are not affected by laser-induced PPTT. The effectiveness of the photothermal treatment relies on the metal surface’s high electromagnetic fields to convert the energy from the absorbed radiation into heat that damages the cells. PPTT is less harmful to human tissue than traditional chemotherapy used by cancer therapies. In general, it is beneficial to use nanorods for therapeutic PPTT whose entire band of absorption can be altered to the electromagnetic spectrum NIR region because of the more substantial visibility of the tissue in this wavelength system. Provided that the plasmon band’s surface wavelength resonates with the gold nanostructures and can be precisely calibrated to their signature measurements, GNR may be promising, especially when functionalized with an immobilized precise homing agent on the particle surface.

**Figure 8:**
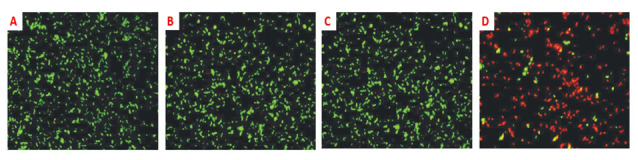
Viability of Pseudomonas aeruginosa cells with attached gold nanorods following exposure to near-infrared (NIR) light. Left panels: (**A**) Cells without nanorods exposed to NIR; (**B**) Cells with nanorods and no NIR exposure; (**C**) (**D**) Cells with nanorods and exposed to NIR for 10 mins. Cells were stained with SYTO 9 and propidium iodide and imaged at 400× magnification using a fluorescence microscope. Green fluorescent cells are representative of live cells while red fluorescent cells are representative of dead or compromised cells. [67]. Reprinted with permission from ref. [67]. Copyright 2011 Wiley Interdisciplinary Reviews—Nanomedicine and Nanobiotechnology.

**Figure 9:**
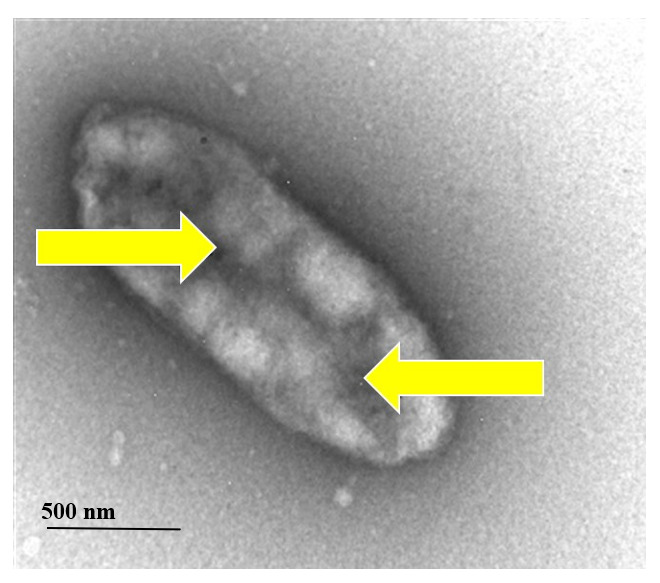
The TEM image of *Pseudomonas aeruginosa* was irradiated at 785 nm for 10 min. The figure evident harm to the bacterial cell, as seen with arrows [67]. Reprinted with permission from ref. [67]. Copyright 2011 Wiley Interdisciplinary Reviews—Nanomedicine and Nanobiotechnology.

In addition to cancers, PPTT was also applied for other treatment purposes. Norman et al. covalently fused massive conjugation of antibodies to gold nanorods to selectively kill *Pseudomonas aeruginosa*, the pathogenic Gram-negative pathogen [63]. Gold nanoparticles can be used for direct conjugation with bio-molecules such as antibodies and other biomolecules. Gold nanorods have been stabilized, conjugated to antibodies, and characterized for biological applications. The stabilizing surfactant bilayer which surrounds gold nanorods was replaced by thiol terminated methoxy poly(ethylene glycol) so that the nanorods are stable in buffer solutions free of surfactant. Nanorod bioconjugation was accomplished with a heterobifunctional cross-linker, with antibody activity confirmed by a strip plate assay. Independent measurements of nanorod chips and antibodies determined the biological composition of the nanorods. Being exposed to NIR radiation resulted in a substantial 75% reduction in bacteria cells’ viability, as shown in (Figure 8 and Figure 9). This work allows for a clear solution to bacterial degradation, which is clinically significant. The researchers applied nanorods to a biological medium and provided sample control tests that were not irradiated to check the cell viability. This work may be further enhanced by adding nanorods conjugated with non-specific antibodies to highlight this method’s selectivity. Black et al. showed optional representation and, following degradation of delta-opioid receptor-expressing cells, using deltaorphin-functionalized GNR, a ligand with a strong affinity delta-opioid receptor [64,65]. Cells that did not produce the receptor were left unharmed while subject to slight irradiation. The investigators have clear proof of cancer cell death by irradiation from the photothermal gold nanorod. Since the initial displacement of CTAB is found by using thiol-containing ligands, further analysis of the ligand-m modified GNR will be needed. As long as the killer cells are not irradiated, a small amount of study will still require. Tong et al. studied the causes and existence of the photothermal damage caused to cell-surface receptors by GNR [66]. The research indicates that the gold nanorod’s photothermal activity goes beyond mere hyperthermia and may interfere with other factors that trigger observed blebbing, such as cavitation and calcium inflow.

As a consequence of photothermal radiation, the investigators provide a fascinating mechanistic theory of cell death. It is not evident whether the nanorods were subjected to a physiological condition like PBS or media before inoculation. Still, it can be seen as having a biological meaning in general. Additionally, the connection to a ligand that is not produced through the folate receptors located on the cell’s surface will further reinforce the claim that the receptor is guided by particle interaction.

**Figure 10:**
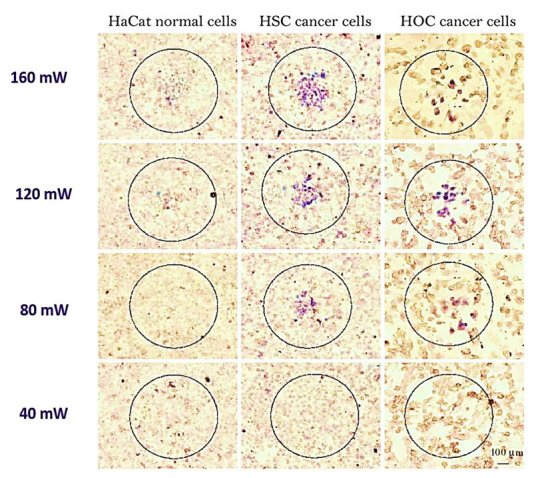
Optional photothermal therapy of incubated cancer cells with anti-EGFR/Au nanorods. The circles on the illustrations show the laser spots [70,77]. Reprinted with permission from ref. [70,77]. Copyright 2006 Journal of the American Chemical Society.

**Figure 11:**
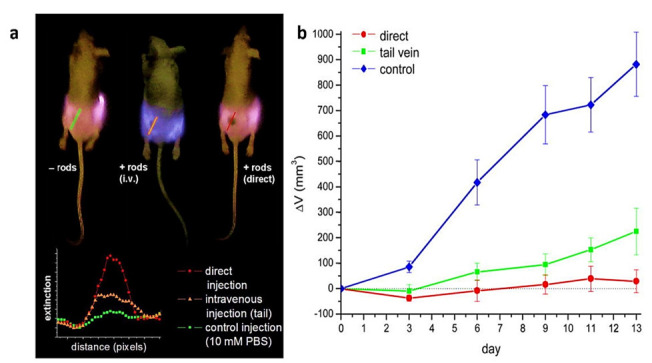
(**a**) NIR transmission images of mice before PPTT treatments. Inset shows intensity line-scans of NIR extinction at tumor sites for control (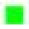), intravenous (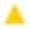), and direct (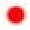) administration of pegylated gold nanorods. Control mice were interstitially injected with 15 μL 10 mM PBS alone. In contrast, directly administered mice received interstitial injections of 15 μL pegylated gold nanorods (ODλ = 800 = 40, 2 min accumulation), and intravenously administered mice received 100 μL pegylated gold nanorod (ODλ = 800 = 120, 24 h accumulation) injections. (**b**) Average change in tumor volume for HSC-3 xenografts following near-infrared PPTT treatment by control (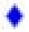), intravenous (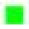), and direct (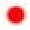) injection of pegylated gold nanorods. Errors for control (n = 10), direct injection (n = 8), and intravenous injection (n = 7) groups were reported as the standard error of the means. Control mice were treated by interstitial injection of 15 μL 10 mM PBS alone. At the same time, intravenous PPTT treatments were performed by administering 100 μL pegylated gold nanorods (ODλ = 800 = 120, 24 h accumulation) followed by 10 min of 1.7–1.9 W/cm^2^ NIR laser exposure. Direct PPTT treatments were performed by administering 15 μL pegylated gold nanorods (ODλ = 800 = 40, 2 min accumulation) followed by 10 min of 0.9–1.1 W/cm^2^ NIR laser exposure [71,77]. Reprinted with permission from ref. [71,77]. Copyright 2008 Journal of the American Chemical Society.

Because of the EGFR’s overexpression on malignant cells’ surfaces, combining monoclonal antibodies with anti-EGFR will destroy malignant cells, specifically attacking the EGFR molecular marker with less than half the laser intensity needed to kill normal cells. Despite this, light penetration of the tissue is shallow (less than 500 μm) at this wavelength [68,69]. While this could help superficial lesions, it is important to have deeper tissue penetration for in vivo cancer treatment. The NIR portion of the spectrum prepares optimum light penetration due to comparatively less dispersion and absorption from the chromophores found in the tissue. Depending on the type of tissue, the light penetration depth in that area is as high as 10 cm. Nanorods are selected with an aspect ratio of 3.9 for absorption of in vitro plasmonic PPTT corresponding to the area of minimal extinction of human tissue. The GNR absorption band also overlaps the Celsius per Watt (CW) Ti: Red laser sapphire at a wavelength of 800 nm. As already mentioned, the laser focuses on falling to the cells treated with a nanoparticle. The cells are dipped in a PBS buffer for 4 min, then laser-exposed. The cells are painted in trypan-blue to check their viability after irradiation. Dead cells absorb the colors that blue the cells, while the dye molecules resist live cells and remain colorless (Figure 10). Normal and different laser energies irradiate cancer cells. Red laser exposure has caused photo destruction of all standard HaCat cells at 800 nm and above 160 mW (20 W/cm^2^) [70]. The malignant HSC cells experience a photothermic lesion at lower laser power. Cell death arises at and above 80 mW in laser spots, corresponding to 10 W/cm^2^. The threshold of death for HSC cells is around half the non-malignant HaCaT cells required to cause cell death. HOC cancer cells also experience photothermal killing at and above 80 mW, although there is no death in the lower-power cells. In this study, all cancer cells consume less than half of the energy needed to kill non-malignant cells. The EGFR molecules expressed over cancer cells and the correspondingly larger anti-EGFR antibody-conjugated GNR attached to EFG receptors. Therefore, these GNR-conjugated antibodies absorb light and turn it into heat on the cell surface and eradicate the cancer cells, while normal cells that do not have these receptors do not develop hyperthermia. The in vitro results suggest that a low-energy, safe, near-infrared laser may be used for cancer cell therapy as a specific and effective photothermal agent. Therefore, the tumor tissue is predicted to be selectively killed by laser energies for further in vivo applications. However, this does not affect the normal tissue surrounding it, owing to the greater concentration of nanorods specifically attached to the tumor tissue. In a mice experiment, therapy is shown as the practicability of near-infrared PPTT in vivo with multifunctional GNR [71]. Subcutaneous xenografts of the squamous cell carcinoma progress in nude mice. MPEG-SH (PEG5000) is conjugated to GNR to develop biocompatibility [55,72,73,74], remove immunogenic reactions, and reduce adsorption of blood vessel’s negative luminous surface. One hundred microliters of PEGylated GNR (OD = 800 = 120) are inserted into the tail vein, and an increased tumor permeability and retention effect (EPR) results in preferential deposition of PEGylated GNR in tumor tissue [65,75]. Silver staining is found 24 h after administration to improve the accumulation of nanorod in the tumor. Then, 15 μL of PEGylated GNR (ODλ = 800 = 40) are injected directly into the tumor interstitium. Tumour administration sites are filled with 10 mM of PBS. Using a thin, lightweight, low-cost, 808 nm (Power Technologies) continuous-wave laser diode, near-infrared PPTT is achieved extracorporeally. In the group in which the nanoparticles were injected intravenously, the mice were kept for 24 h to maximize the accumulation of GNR in the tumor cells, followed by NIR radiation with an intensity of 1.7–1.9 W/cm^2^ for 10 min. In the group in which the nanoparticles were directly administrated into the tumor, the mice were extracorporeally irradiated by NIR radiation with the same intensity and time. Accumulation of nanorods is managed by NIR transmission imaging following direct and intravenous administration (Figure 11). NIR extinction force line scans revealed limited diffusion for more than 3 min of directly injected particles, with no further production for several hours. NIR-transmission line scans of HSC3 tumor sites, injected with 15 μL of 10 mM PBS, indicate partial extinction leading to excessive tissue density. In comparison, line scans attained after 24 h accumulation of intravenous injection showed almost three times the extinction observed for control sites. The NIR extinction after direct intratumoral injection of GNR was shown to be more than twice that of intravenous administration and more than seven times that of control cells. The average tumor volume rise, reported over 13 days (Figure 11), indicates a decrease of >96 percent in normal tumor growth for directly treated tumors and a decrease of >74 percent in the medium development of intravenously treated HSC-3 xenografts at day 13 (towards tumor control). Resorption over the measuring period of >57% of tumors treated directly and 25% of tumors treated intravenously was also reported. In comparison, none of the control tumors experienced either growth suppression or resorption. The heating output for PPTT treatment was 3.59 ± 0.5 for direct injection and 1.90 ± 0.4 for intravenous injection of PEGylated GNR. Heating output was defined as the ratio of changes in the steady-state temperature in the presence and absence of plasmonic particles. The former value was in accordance with the study of Hirsch et al. reported in vivo PPTT treatments with near-infrared [76]. They inject nanoshells directly into the gold. The observed variations in temperature change by direct and intravenous administration for control treatments correspond well with power density variations. The difference in direct and intravenous PPTT heating output is induced by intravenous injection at a proportionally reduced particulate charge, in accordance with the straight-line scans obtained from NIR transmission images. Although the volume and concentration of particles for intravenous injections are significantly higher, the degree of angiogenesis of the tumor and the reticuloendothelial system (RES) likely limit the aggregation. Treatment selectivity and effectiveness for direct injections were more evident than other methods; nonetheless, both approaches have demonstrated dramatically increased local tumor control.

Multifunctional GNR-PPTT against cancer and bacterial cells has been performed utilizing in vitro and in vivo models [57,70,77,78]. A negligibly obtrusive therapy strategy may be the photothermal solution for inducing hyperthermia to the tumor cell. Often in PPTT, multifunctional GNRs are employed as “theranostic” dealers; this is indeed one of the self-evident aims of biomedical nanorod research. As described above, the NIR mellow GNRs have a more appealing and prominent tunable retention band relative to different gold nanoparticles; for heat treatment, the light is expected to warm enough to the GNR. Notable occlusion and warm elimination of tumor fractions were observed after orchestrated light and intravenous injection into mice of PEG-modified GNR [79,80]. Huang et al. have proposed [70]. The use of GNR for cancer cell slaughter as photothermal restorative specialists. GNR in cancer cell lines has been conjugated with monoclonal antibodies to recognize over-expressed proteins. By contrast, the GNR brooded a non-malignant epithelial cell line, and malignant epithelial cell lines inserted xenografted tumors into nude mice using adapted GNR with polyethylene glycol (PEG). The tumors were lighted at this stage with an 810-nm laser if the temperature had increased to 70 °C after 5 min.

 **(a)**  **Cancer Therapy**

**Figure 12:**
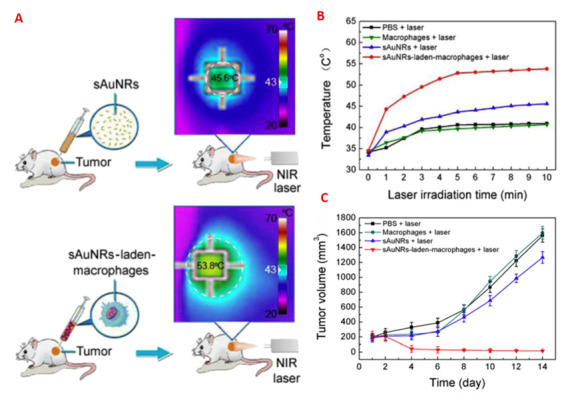
(**A**) Schematic showing the distinction between the treatment of small free gold nanorods and small gold nanorods charged with macrophages; (**B**) tumor temperature profile below 808 nm light for 10 min; and (**C**) tumor growth after irradiation therapy in different mice classes [42,84]. Reprinted with permission from ref. [42,84]. Copyright 2007,2016 Biosensors and Bioelectronics.

To resolve the limitation that the nanoparticles injected cannot penetrate the tumor mass resulting in insufficient removal and recurrence of the disease [74], cell-mediated distribution of nanoparticles capable of overcoming nearly impermeable biological barriers in many areas of the body [81,82,83], were proposed to enhance the dissemination of in vivo agents and increase the production of photothermal agents. On this basis, Chu et al. used the process of transmission of macrophages [84] for the transport of Au nanorods 7 nm in diameter for cancer therapy (Figure 12). First, they examined the absorption of macrophages, which is essential for photothermal conversion. The tiny gold nanorods showed much higher macrophage uptake and negligible cytotoxicity due to their small size. Compared with the commonly used 14 nm diameter gold nanorods, the small gold nanorods showed much higher macrophage uptake and negligible cytotoxicity due to their small size. Then, the photothermal therapeutic effect was studied by intratumoral injection of 50 µL of PBS (control), free small gold nanorods (105 µg Au) dispersed in 50 µL of PBS or small gold nanorod-laden macrophages. After intratumoral injection, the macrophages may administer small GNR to the entire tumor, resulting in a dramatic improvement in photothermal conversion nearly anywhere in the tumor, with tumor recurrence levels declining relative to free small GNR coated with BSA. Their results offered an efficient means of improving phototherapy efficiency by spreading the agents to entire tumors and increasing nanotechnology’s clinical application for cancer treatment [42].

**Figure 13:**
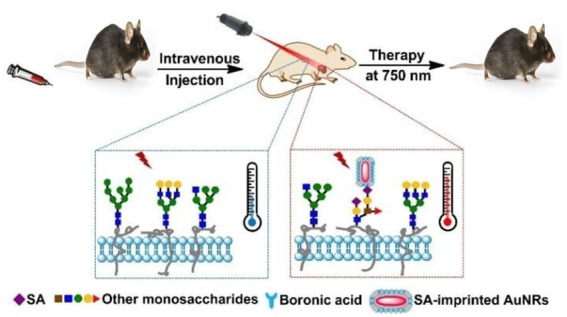
Demonstration of the principle for targeted photothermal therapy through SA-imprinted GNR @SiO_2_ [85]. Reprinted with permission from ref. [85]. Copyright 2017 ChemComm.

As proof of the principle, GNR was used as the central plasmonic nanomaterial. In contrast, Sialic acid (SA) was used as a template for Macrophage inflammatory protein (MIP) preparation and targeted cancer cell identification. The principle of cancer-based phototherapy is described in (Figure 13). SA-printed GNR is inserted intravenously into a tumor-bearing mouse. GNR disperse together with the bloodstream within the animal. GNR accumulates exclusively in tumors due to sensitivity to SA. The NIR laser beam (750 nm inside) is guided at the tumor for a certain length. The SA-imprinted GNR consumes the NIR light’s photon energy and transforms it efficiently to heat. Therefore, the heat produced by the GNR explicitly kills the tumor and leaves the healthy tissue around it undamaged. Specific PTT therapies will fully ablate the tumor. This gives a new perspective on targeting PTT cancer.

 **(b)**  **Antibacterial Activity**

Gold nanorod (GNR) suspension Antibacterial activity of gold nanorod suspension (GNR) with specific surface yield characteristics against standard strains of *Staphylococcus aureus* and *Propionibacter-rhymes acne* was investigated. The colloidal integrity of GNR suspensions is associated with bacterial growth media and the potential presence of impurities from GNR suspension. Findings revealed that cationic polyallylamine hydrochloride (PAH)-GNR was strongly aggregated relative to other GNR suspensions when presented to bacterial development media. Furthermore, CTAB is most definitely the cause of the antibacterial behavior found in GNR suspensions. Nevertheless, GNR’s antibacterial role itself could not be ruled out. Preparing these two necessary monitoring tests prevents the antibacterial action from misinterpreting nanoparticles and artifacts. Unfortunately, some approaches are generally ignored in published studies, which can clarify the most contradictory results. The study also reveals that GNR can be a practical treatment choice for follicular skin diseases such as acne vulgaris [86,87,88,89].

## 6. GNR in Novel Pharmaceutical Delivery Systems

 **(a)**  **Drug Delivery Vehicles**

In cancer, the main problem is the mechanisms of professional nanoparticle release. For example, heat transferred from standard material can be combined with GNR to effectively deliver drugs to metal nanoparticles [90], enhancing phototral effects. GNR can discharge biological products and molecules in Yamashita nano thermal devices [91,92,93]. In 2011, PEG-linked Diels–Alder cycloadducts on GNRs were altered to shape a controlled-release structure triggered by retro Diels—GNR suggested the photothermal effect-induced alder reaction. Diels–Alder’s solution is commonly used as a reversible solution to cycloadding to create a cycloadduct between the alkenes and the diene firms. Due to the nature of retro Diels–Birch reaction, PEG chains were thrust through GNR or warm treatment after laser illumination from the gold nanorod surface. PEGylation ensures the GNR can be covered with a thermo-sensitive shell that disperses sedated particles in controlled dispatch. The photothermal effect of gold nanorods actuated by irradiating NIR light induces phase transition of the thermo-responsive polymer [94].

Another example is the polymer-coated GNR, which function as a reservoir of a product with a limited premature release. Then, in the year 2013, Shen et al. [95] modified GNR filled with Doxycycline (DOX) (GNR-Polycaprolactone PCL-b-PEG-DOX), which tend to be used in addition to tumor therapy. This research into chemotherapy and phototherapy tended to be synergy-led. A changed medication is attached to the PEG chain; the controlled-release device should be appropriately identified. It ensures the GNR can be coated with a thermo-sensitive shell that disperses sedated particles. NIR illumination of GNR causes heat, stimulating a region going into the extended form and thus discharges the drug [21].

**Figure 14:**
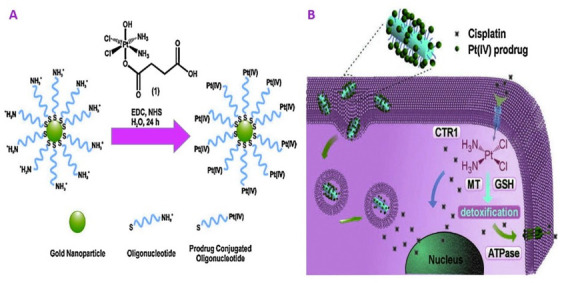
Gold nanoparticles and platinum (IV) nanorods for the delivery of drugs. The cisplatin platinum (IV) prodrug was conjugated through EDC/NHS coupling to gold nanoparticles (**A**) and nanorods (**B**) [97]. Reprinted with permission from ref. [97]. Copyright 2015 Materials Today.

Platinum (IV) prodrugs were also tied to GNR (Figure 14) [96], which further increased platinum uptake, reduced glutathione-mediated detoxification, and ultimately overcame resistance to cisplatin in lung cancers.

 **(b)**  **Gene Delivery**

The simplicity of changing the level of GNR, the possibility of combining biological drugs and molecules, in addition to their various optical and electronic properties, has attracted the most interest in genes and pharmaceutical applications [95,98]. Adjust in fibrous tissues, Surface plasmon resonance (SPR) products may be regulated [52]. Using interfering RNA is a frequent and commonly established gene silencing technique to prevent protein synthesis by hybridizing short interfering RNA (siRNA) [99]. However, methods for the transmission of siRNA and the control of spatiotemporal operation remain a concern. Bonoiu et al. proposed that GNR may be used to modulate important dopaminergic signaling pathway components with small interfering RNA (siRNA) molecules [100]. To check the penetration of siRNA-conjugated GNR neuronal (DAN) cells in vitro dopaminergic by using cell lysate fluorimetric analysis. They witnessed the DARPP-32 gene being suppressed within DAN cells. They demonstrated that the siRNA conjugated GNR in vitro exhibited substantially higher transmigration efficiency through the blood-brain barrier (BBB) relative to free siRNA, without weakening. The authors previously used fluorescent-labelled siRNA against glyceraldehyde 3-phosphate dehydrogenase (GAPDH)-functionalized GNR to suppress target gene expression in the rat hippocampal CA1 without cytotoxicity [101]. These reports are essential because the brain is an especially distant drug delivery organ. Because the brain is one of the special organs for drug delivery due to the presence of BBB [102], it opens up new treatment options for neurological disorders or brain tumors. Together with the enhanced protein of green fluorescence (EGFP DNA), GNR regulates gene expression in living cells [103]. Based on this property, the GNR will change their shape after NIR photons are absorbed [104]; the NIR illumination of the EGFP DNA-conjugated GNR resulted in a shape transformation, which in turn led to the release of EGFP DNA. GNR encapsulated in graphene oxide nanosheets demonstrated excellent efficiency in delivering DNA to HeLa cells utilizing another technique [105,106]. The multifunctional framework showed how chemotherapy and the silencing of RNA were combined. Conjugated poly (amino ether) GNRs have been used to deliver short hairpin RNA (shRNA) plasmids to silence the luciferase gene expressing the luciferase protein that is constitutively expressed in prostate cancer cells [107].

Similarly, polyamidoamine dendrimer-coated GNR showed high efficiency in delivering the brcaa1-shRNA gene into the MCF-7 breast cancer cell, which has significant inhibitory activity on MCF-7 cell (MCF-7 is the acronym of Michigan Cancer Foundation-7, referring to the institute in Detroit where the cell line was established in 1973 by Herbert Soule and co-workers) growth [108]. In addition, Lee et al. developed and implemented photonic gene circuits utilizing optically-addressable siRNA-AuNR nanoantennas in living cells [109]. The authors pick GNR as nanoantennas in the NIR system, where cells are significantly transparent due to their broad optical absorption cross-section, longitudinal plasma resonance band wide spectral bandwidth, and tunable longitudinal plasma resonance wavelength. GNR can be used in phototherapy and phototherapy because it acts as a controller of the mechanism for the secretion of drugs that cause NIR radiation.

**Figure 15:**
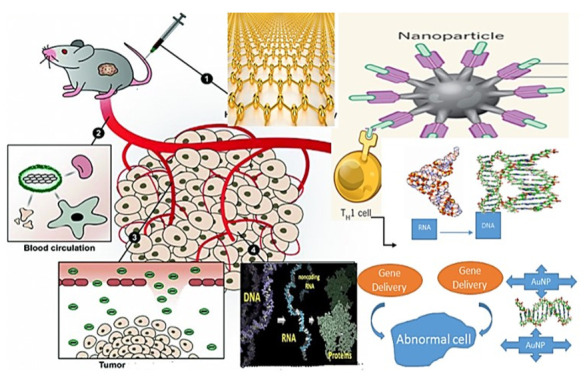
Schematic Gene Delivery of Gold Nanorods application.

**Figure 16:**
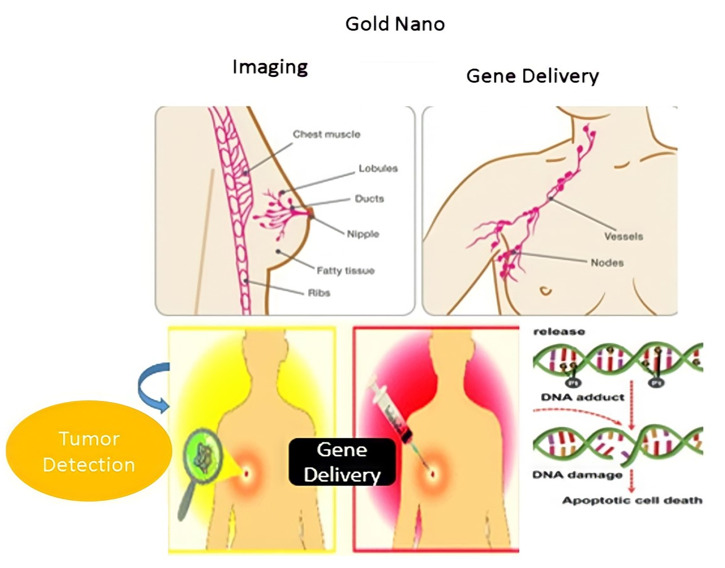
Schematic Gene Delivery of Gold Nanorods within DAN for MCF-7 breast cancer cell application.

Nanoscale GNR Critical objects are critical to clinical diagnosis. The physical and chemical properties of GNR therapy, including its size, shape, and surface characteristics, are important factors influencing their cytotoxicity. The photothermal effect revealed that double-stranded GNR (dsDNA)-modified by NIR light released the single-stranded DNA (ssDNA) from the gold nanorod surface. The sum released depended on the influence and displayed time of the irradiation [92,110]. The effect was also documented for single-stranded oligonucleotide-coated GNR **(Figure 15 and Figure 16**) [111].

## 7. GNR Cellular Metabolic Responses and Adverse Biological Effects

Nanoscale GNRs are essential properties that have important properties, including their size, shape, and surface characteristics, influencing their cytotoxicity. Although the bulk gold is deemed “safe”, the biocompatibility and environmental effects of GNR must be examined. Literature review shows many academic types of research show the effect of various aspect ratios and surface modifications on the cytotoxicity and cell absorption of GNRs by cultured cells. The findings showed that surface chemistry controls the biological toxicity but does not affect the GNR factor ratio. CTAB-GNR can destroy mitochondria with specific aspect ratios and stimulate intracellular reactive oxygen species (ROS) to cause cell apoptosis and autophagy. Intravenous injection of CTAB/PAH GNR causes GNRs to enter tumor tissue through the bloodstream of animals. It remained constant and the other half-life of the GNR was longer. Wan et al. observed that more coating on the administration of CTAB-coated GNR could prevent cytotoxicity and cell death and change the GNR aspect ratio and demonstrate more considerable biological possessions with improved biocompatibility and minimal cytotoxicity [112].

MTT assay with various cell lines shows that the CTAB-capped GNRs were confirmed to be cytotoxic. PEG minimizes GNR cytotoxicity, which is well known to restrict unspecific surface attachment to biological molecules. Analogous cellular uptake studies showed that the PEG-coated GNR uptake was 6 percent compared to the original GNR coated CTAB [113]. Another biocompatible overcoating agent, phosphatidylcholine, reduces the reported cytotoxicity of CTAB-coated GNR. Deshani et al. observed that GNR covered with CTAB and then overcoated with polyelectrolytes show 90 percent cell viability for HeLa cells and cause no change in gene expression levels: only 35 of the 10,000 genes analyzed were slightly down-regulated [114].

In addition to cytotoxicity, specific pharmacokinetic experiments such as biodistribution and metabolism problems need to be discussed for biomedical applications, which, in the case of GNR, has thus far gained less study. This method requires accurate molecular information about the unique intracellular position, cellular absorption, and translocation of GNR to normal and tumor cells, highlighting the significance of metabolomics in determining the biological effects of GNR. The cell’s local mechanical environment is part of a dynamic feedback loop regulating cell metabolism, gene expression, and migrations.

Analysis of the cell lines found that GNR translocated from the lysosome to the mitochondria in most cases but not in all cell lines. Nonetheless, during this cellular translocation, the molecular details remain completely undetermined [115]. Additionally, GNR mediated cell death in the intracellular environment, which was reflected metabolically in lactate levels. GNR also caused oxidizing tension in cell lines. Extreme oxidative stress causes damage to mitochondria resulting in cell death, which is evident in the pronounced decrease in nucleoside levels and nucleotides. Besides, considerably increased amino acids are likely due to the stress hormones released in GNR-treated cells [115].

Although the cytotoxicity analysis did not lead to substantial declines in cell proliferation by GNR-PEG and GNR-Mercaptohexadecanoic acid (MHDA), the expression of stress- and toxicity-related genes was substantially impacted. These gene expression improvements were more critical for GNR-MHDA, which can be attributed to the higher degree of association with serum proteins in the biological media that are likely to be actively shared with various macromolecules until incorporated into the cell environment. In contrast, GNR-MHDA showed a significant interaction with cell membrane uptake for GNR-PEG. If shifts in gene expression continue for prolonged use, the toxicity for chronic GNR-MHDA use is possibly higher [116].

An in vivo study by Li et al. shows the reactivity of Au@Ag NR to inflammation, followed by considerable alterations in metabolism. Based on metabolic profiling, they found changes in dopamine synthesis, redox metabolism, purine metabolism, energy metabolism, choline metabolism, and membrane [117]. The enhancement of the dopamine metabolism on non-neural tissues was first observed and connected with inflammation. The inflammation and injury are demonstrated by histochemical testing of the liver and lung. Inflammatory cells can reside in blood at the periphery. The increase in IL-1β and IL-6 levels following treatment with Au@Ag NRs indicated inflammation [117]. Since such results could not be entirely attributed to GNR, questions regarding the in vivo harmful effects of these intriguing nanorods may escalate more than ever before and should not be restricted to a simple test of the toxicity studies suggested. Another research showed that GNR, which is treated positively or negatively, has various metabolic implications: insignificant cell metabolism disturbances, the cytotoxicity of negatively charged GNR mediated energy metabolism, choline metabolism, hexosamine biosynthesis process, and oxidative stress of cells.

Although the metabolome is more similar to phenotype, it may be suggested that metabolomics be used to understand the metabolic effects of GNR better until they are used for clinical applications.

## 8. GNR in Oxidative Stress

Despite the growing use of PPT as an efficient cancer therapy, the interaction between chemically induced cell death and thermally induced cell death during the PPT treatment via the formation of reactive oxygen species (ROS) has not been surveyed well. Oxidative stress also has an essential role in cancer, Rheumatoid arthritis, and Alzheimer’s [118,119]. ROS production rates and cellular sites during temperature stress could play a central role in stress perception and protection [120,121]. The endocytosis of spherical NPs by cells is comparatively less poisonous and is relieved more than rod-shaped NPs. Some outcomes contradict each other, and few strong tendencies of information have been determined so far. For example, as they can be the reason for breast cancer cell death because of their shape, among other causes, GNR displays excellent potential in cancer hyperthermia than usual spherical gold nanoparticles [122,123]. This outcome has happened because of the lack of an adequate sample for measuring these relevant attributes [124,125,126,127,128]. The impact of this issue is their impact on the biological method and the remarkable properties of the materials. In this part, the affectations of GNR physicochemical attributes, such as charge, surface chemistry, shape, size, and other factors on the biological system, are reviewed.

 **(a)**  **Effect of Surface and Shape for the Cellular Response**

In cytotoxicity, dose uptake is one factor, and a key issue for toxicological research is cell uptake [129]. Thus, in cytotoxicity, uptake of dosage is one of the agents. For nanoparticles (NPs), many parameters can influence cellular uptake. However, the intracellular uptake mechanism is not apparent, and the outcome has not been achieved. Various investigations have taken place to survey the surface physicochemical attributes that can affect cellular uptake. For example, in cellular uptake, the surface charge has a considerable role, unlike other factors [130,131]. Rapid sorption of serum showed the actual situation of the GNR negatively charged serum-coated appearance before uptaking. However, all GNRs offer similar potential to Zeta.

Protein uptake can affect cell uptake, which discovers the functional diversity of surface-absorbed proteins [132]. Maybe the value of adsorbed proteins is one variation among these GNR. NPs comfort the transmembrane internalization and identify the membrane receivers because, on the surface of NPs, more proteins exist, and they may have better feasibility to subject ligands. Using reducing SDS-PAGE, the value of protein adsorbed by GNR was measured. Protein adsorption on GNR achieves the equilibrium more quickly [133]. Protein uptake in GNR is rapidly balanced. The minimum desirable poly (diallyl dimethyl ammonium chloride) PDDAC-coated GNRs adsorb the least amount of proteins, and more desirable PDDAC-coated GNRs in cellular uptake adsorb more quantity of proteins.

 **(b)**  **CTAB: The Real Reason for Cytotoxicity for GNR**

**Figure 17:**
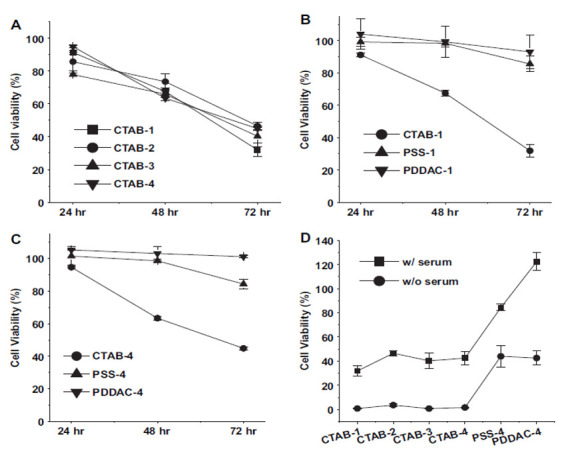
Cytotoxicity of treated cells by Au NRs. (**A**) cholecystokinin CCK-8 assays of CTAB-coated Au NRs (**B**) AR ¼ 1 in Au NRs (**C**) AR ¼ 4 in Au NRs (**D**) CCK-8 assays of Au NRs [137]. Reprinted with permission from ref. [137]. Copyright 2010 Biomaterials.

GNRs were found to be toxic to cells, so a double layer of CTAB molecules coated on the surface of the synthesized GNR [113,134,135]. Nevertheless, the cytotoxicity of GNR reductions was considerable, with major covered negatively charged PSS (Figure 17B,C), which is in agreement with previous investigations [131,134,136].

**Figure 18:**
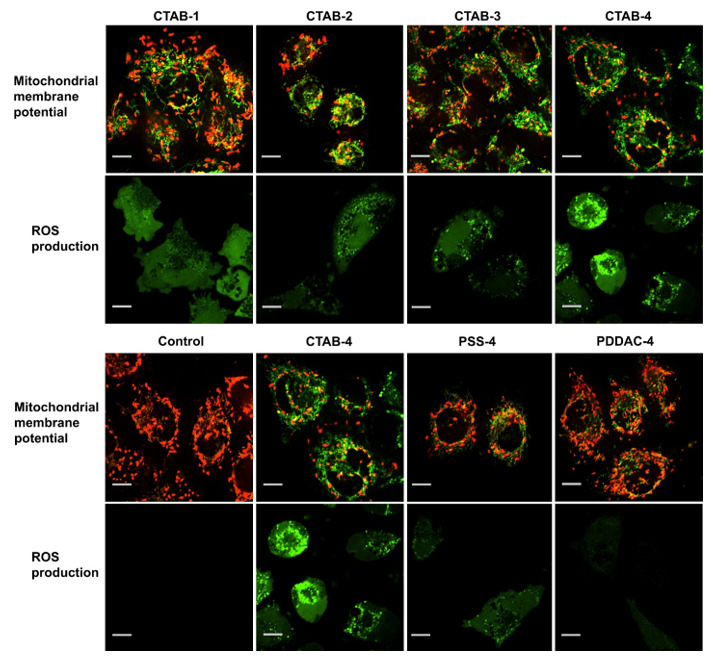
GNR of different coatings or ARs. JC-1 (red fluorescence), ROS level (green fluorescence), causing mitochondrial damage [137]. Reprinted with permission from ref. [137]. Copyright 2010 Biomaterials.

In addition, a large amount of coating has a positive charge of PDDAC, and this positive charge weakly reduces the toxicity of GNR reduces the toxicity of GNR to a poor rate. Even with the lack of newborn calf serum in the culture medium, the PDDAC and PSS-coated GNR show very high cell growth rates (Figure 1). The ROS rates in MCF cells treated with PDDAC or PSS-coated GNR and the mitochondrial membrane potential show lesser changes (Figure 18). Thus, we can conclude that CTAB molecules have an essential role in the cytotoxicity of GNR.

**Figure 19:**
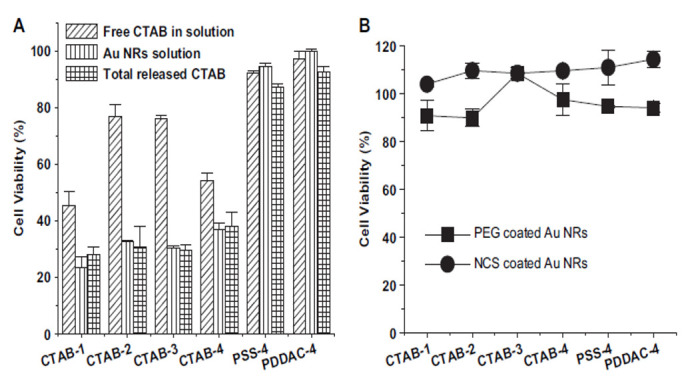
Cell viability after treatment with (**A**) supernatant of Au NRs suspensions and PEG. (**B**) Au NRs are coated with newborn calf serum or PEG [137]. Reprinted with permission from ref. [137]. Copyright 2010 Biomaterials.

**Figure 20:**
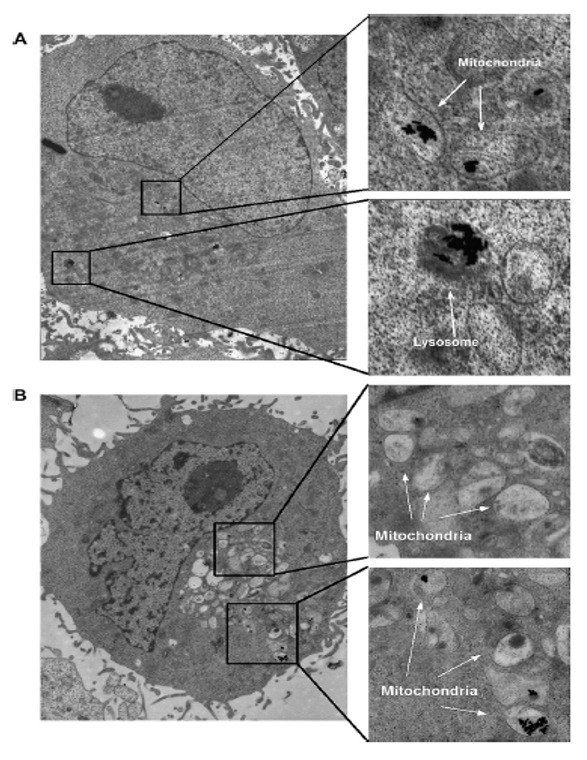
TEM images of (**A**) Au NRs in lysosome or mitochondria (**B**) Cells with CTAB molecules [137]. Reprinted with permission from ref. [137]. Copyright 2010 Biomaterials.

Even if all GNRs in the laboratory are washed twice, the GNRs have a substantial CTAB value. Extreme washes cause severe aggregation of the GNR in the lack of CTAB from the rod surface because the binding between GNR and CTAB is not as powerful [130]. The source of CTAB-coated-GNR solution was centrifuged. After gathering the supernatant, it was found that it is toxic for cells but in a lesser amount than GNR (Figure 19A). By reproducing CTAB from the GNR surface through a ligand replacement, we found the cytotoxicity of total CTAB. Then, 100 times excess carboxyl PEG-SH was mixed with GNR suspension and sonicated. The achieved PEG-coated GNR display poor cytotoxicity (Figure 19B), which represents an almost complete substitution. To measure the cytotoxicity by the CCK-8 method, the final supernatant was utilized, CTAB, one of the cations and cationic surfactants. Surprisingly, a great can be found for the cell’s livability between treated cells with CTAB-coated-GNR, and these supernatants treated cells (Figure 20A). The result shows that due to CTAB molecules, CTAB-coated GNRs are cytotoxic but not from the GNRs themselves.

 **(c)**  **Mitochondria Dysfunction of CTAB-Cause for Apoptosis**

Mitochondria dysfunction in subcellular rate is done by CTAB, which is one of the cations and cationic surfactants. Inflammatory responses of mitochondria are shown by TEM images (Figure 20B), which include vanishing of cristae, swelling in volume, and enhancing in number.

The swelling of mitochondria is a marker of outer membrane rupture, and the cumulation of the GNR in cells causes organelle edema [138]. Mitochondrial membrane potential (ΔΨm) has an essential role in the electron transition chain and retains the proton slope through the inner mitochondrial membrane. Any infraction over it may cause inducement problems in many metabolic procedures, such as cell cycle regulation, ATP synthesis, and maintenance of mitochondrial integrity. Cells treated by CTAB-coated GNR indicate a fundamental variation in ΔΨm in comparison with control, suggesting distinct mitochondrial damage and depolarization. Vice versa, in cells treated with PDDAC or PSD-coated GNR, lesser injuries are observed.

This matter is attractive despite their major primary uptake capability and poor toxicity, especially for PDDAC-coated GNR. There must be sufficient free CTAB in the suspension of CTAB-coated GNR to retain the dispersion. However, the condition is quite different in the suspension of PDDAC or PSS-coated GNR. After polyelectrolyte coating of GNR, all free suspended CTAB was moved by centrifugation, and polyelectrolyte could stabilize the GNR. The polyelectrolyte coating releases the encapsulated CTAB molecules that cannot break. Thus, the PDDAC- or PSS-coated GNRs investigate minor toxicity because the polyelectrolyte coating to release the encapsulated CTAB molecules cannot fail. Without the suitable subordinate to reduce the artificial polyelectrolyte, the enzymes in lysosomes cannot break the coatings without the suitable subordinate to reduce the artificial polyelectrolyte.

 **(d)**  **The Cytotoxicity Mechanism and Complete Procedure of GNR Uptake**

**Figure 21:**
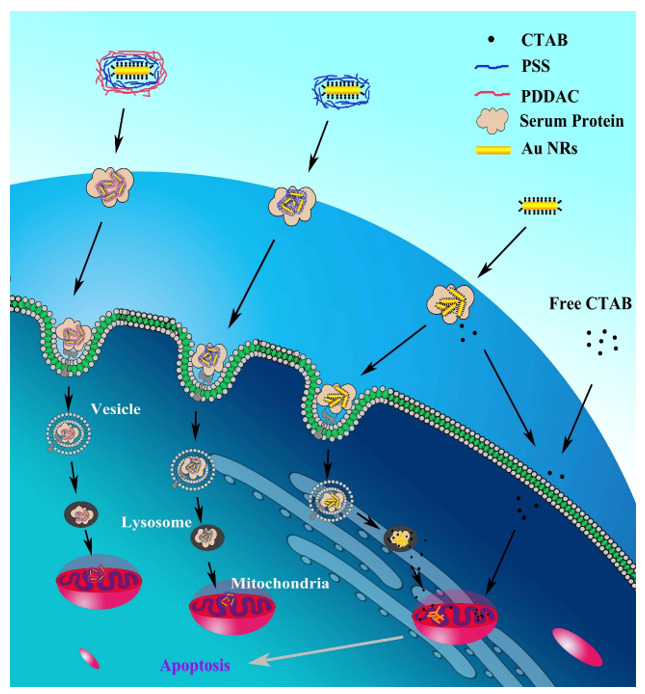
The mechanism for cytotoxicity and GNR uptake [137]. Reprinted with permission from ref. [137]. Copyright 2010 Biomaterials.

The mechanism for cytotoxicity and GNR uptake has been shown in Figure 21. In summary, GNRs are quickly coated by serum proteins after being added to the culture medium. The protein coatings cause interference of cellular uptake, likely via receptors caused by the formation of small aggregates. GNR can be transported and accumulated in mitochondria due to coating with some other proteins [137].

## 9. Prospects for the Future

GNR is an enticing substitute to conventional organic fluorescent colors because they do not photo bleach, diffuse in the transparent and NIR, and are non-toxic in some laboratory circumstances. It seems that intensive research will be carried out over the next several years, based on GNR’s in vivo tests. There is also a fair volume of the literature on in vitro diagnostics. For very different subject cells (e.g., certain malignancies, bacteria), most visualizing operations will be linked to a particular diagnosis, testing, and care. As a function of the size, form, and surface coating of nanorods within the primary cell lines, toxicity and side effects must also be carefully studied for each sample until human subjects can be subjected to these products.

We believe that more insight is needed into how cells and organs work. Suppose the biomedical experts accept GNR as a modern material for in vivo imaging, for longer time scales and less history than the existing fluorescent samples. In that case, we believe that more insight will be gained on how cells and organs work with others internally and externally. It would appear that expanded collaboration among researchers in biology, medicine, nanoscience, and nanotechnology will provide new fundamental insights into biological processes.

## 10. Conclusions

GNR appears to be promising materials for drug delivery and photothermal therapy. The ease with which the materials may be synthesized in water, their degree of size and shape control, and hence optical property control, and the capacity to alter the surface chemistry are all advantages for biomedical applications. Several methods are used to synthesize GNR, among which the bottom-up method is considered a very effective method for the synthesis of nanorod particles. However, the top-down approach is time consuming and costly for the mechanical part. We summarized that the efficacy of nanorods is unambiguously dependent on the component nanorod proportion, degree division, polydispersity, and implementation. Despite the promising future of GNR in biological fields, several fundamental challenges need to be addressed. Understanding how gold GNR basic components affect cellular response is critical in moving gold nanomaterials beyond research novelty and into the realm of therapeutic treatment.

The environmental and biological impacts of the chemical and metabolic remnants of GNR-based products were rarely studied. Therefore, the cytotoxicity and cell absorption of GNR by cultured cells and mice was reviewed in-depth. Analysis of the cell lines found that GNR translocated from the lysosome to the mitochondria in most cases but not in all cell lines. Additionally, GNR mediated cell death in the intracellular environment, which was reflected metabolically in lactate levels. GNR also caused oxidizing tension in cell lines. Extreme oxidative stress causes damage to mitochondria resulting in cell death, which is evident in the pronounced decrease in nucleoside levels and nucleotides. Besides, considerably increased amino acids are likely due to the stress hormones released in GNR-treated cells. It seems that intensive research will be carried out over the next several years, based on GNR’s in vivo tests. There is also a fair volume of the literature on in vitro diagnostics. For very different subject cells (e.g., certain malignancies, bacteria), most of the visualizing operation will be linked to a particular diagnosis, testing, and care. As a function of the size, form, and surface coating of nanorods within the primary cell lines, toxicity and side effects must also be carefully studied for each sample until human subjects can be subjected to these products.

## Data Availability

Data available in a publicly accessible repository.
